# Paraneoplastic LGI1 Encephalitis Associated with Lung Adenocarcinoma: A Case Report

**DOI:** 10.3390/neurosci6020043

**Published:** 2025-05-15

**Authors:** Francesco Rossato, Andrea Porsio, Diego Cecchin, Matteo Atzori, Anna Maria Basile, Marco Zoccarato

**Affiliations:** 1 Neurology Unit, Ospedale Sant’Antonio, Azienda Ospedale Università di Padova, 35218 Padua, Italy; 2 Nuclear Medicine Unit, Department of Medicine—DIMED, Azienda Ospedale Università di Padova, 35128 Padua, Italy

**Keywords:** LGI1 antibodies, limbic encephalitis, paraneoplastic neurological syndromes, lung cancer, adenocarcinoma

## Abstract

Limbic encephalitis (LE) associated with anti-LGI1 antibodies is an autoimmune disorder characterized by memory decline, behavioral changes, and temporal lobe epilepsy. Faciobrachial dystonic seizures (FBDS) are a hallmark symptom, often preceding cognitive and psychiatric issues. This report presents an 80-year-old male with LGI1 encephalitis, initially manifesting as FBDS. A lung adenocarcinoma was diagnosed two months after the onset of neurological symptoms. Clinical and paraclinical data, including MRI and [18]FDG PET imaging, are described. The patient responded to immunotherapy, including steroids and plasma exchange, along with tumor resection. Following treatment, neurological symptoms resolved, except for mild anxiety and apathy. Further research is needed to determine whether LGI1 encephalitis may occasionally have a paraneoplastic origin, potentially influencing screening and management strategies.

## 1. Introduction

Limbic encephalitis (LE) associated with leucine-rich glioma-inactivated-1 (LGI1) antibodies is an autoimmune disease characterized by a subacute decline in memory, behavioral disturbances, and temporal lobe epilepsy. Faciobrachial dystonic seizures (FBDS) are a hallmark of the disease, particularly in its early stages. These brief and repetitive episodes involve dystonic posturing of the face and arm on the same side, and can occur hundreds of times per day [1].

LGI1 antibodies are the most common anti-neuronal antibodies found in suspected autoimmune encephalitis in patients over 50 years of age [1,2]. They target a protein primarily expressed in the hippocampus and temporal cortex, which plays a key role in an inhibitory pathway linking the presynaptic voltage-gated potassium channel (VGKC) to the postsynaptic α-amino-3-hydroxy-5-methyl-4-isoxazolepropionic acid receptor (AMPAR) [3]. Consequently, most patients develop a clinical presentation consistent with LE, while a smaller subset may exhibit other manifestations, such as Morvan syndrome (LE with peripheral nerve hyperexcitability and insomnia/dysautonomia) or isolated epilepsy [4,5]. The trigger of the autoimmune response remains unknown in most cases of LGI1 encephalitis, and only a minority of cases are associated with cancer (paraneoplastic LE) [6].

In this report, we present a case of LGI1 encephalitis initially manifesting as isolated FBDS, occurring in close temporal association with the discovery of lung adenocarcinoma. Following a comprehensive treatment approach—including immunotherapy and surgical resection of the tumor—the patient achieved complete resolution of neurological manifestations.

## 2. Case Description

We report the case of an 80-year-old Caucasian man, former smoker, with a history of hypertension, hypercholesterolemia, and benign prostatic hyperplasia. A retired surgeon, he was fully independent in his daily activities. In May 2022, the patient developed brief and recurrent episodes characterized by sudden, predominantly right-sided (although the controlateral side could have been involved) involuntary upper limb movements and loss of handgrip, leading to object-dropping. The patient was unaware of the episodes, which were primarily reported by his wife. There was no clear triggering factor. Electroencephalography showed diffuse sharp theta-wave activity. A working diagnosis of focal-onset epilepsy with impaired awareness was made, and empirical treatment with lacosamide was titrated to 50 mg twice daily, with a partial response. In June 2022, the patient underwent brain magnetic resonance imaging (MRI), which revealed punctate hyperintensities in the deep white matter of the frontal and parietal lobes on T2- and fluid-attenuated inversion recovery (FLAIR)-weighted sequences, consistent with vasculopathy. In July 2022, the patient was admitted to the Emergency Department for acute chest pain. Thoracic computed tomographic (CT) angiography revealed bilateral pulmonary embolism, and systemic anticoagulation with apixaban was initiated. Incidentally, the thoracic study also revealed a left apical lung mass measuring 33 × 28 × 40 mm, with an air bronchogram and intermixed small calcifications. The mass was further evaluated using whole-body 18F-fluorodeoxyglucose positron emission tomography ([18]FDG PET), which revealed hypermetabolism. No additional hypermetabolic foci were detected. In September 2022, a CT-guided biopsy of the pulmonary mass confirmed the presence of non-small-cell lung carcinoma, specifically classified as adenocarcinoma. Following this diagnosis, the patient was scheduled for surgical removal of the tumor in the following weeks.

While awaiting the planned surgical intervention, in October 2022, the patient began experiencing an increase in the frequency of seizures, despite adjustments and escalation of antiseizure treatment. In parallel, he developed episodic memory deficits, which gradually worsened until they culminated in a persistent confusional state. This cognitive deterioration eventually prompted a visit to the Emergency Room. The patient’s wife provided additional details, reporting significant memory impairment over the preceding weeks, particularly in recalling important recent events. One striking example was his inability to remember attending a close relative’s wedding.

Upon admission to the Neurology Ward, physical examination revealed an awake and alert patient, without speech impairment, but he displayed noticeable anxiety, the presence of ideomotor apraxia, and a significant impairment in episodic memory encoding. The raised-arm test uncovered mild dystonic posturing of the right hand, accompanied by bilateral postural and resting tremor. Additionally, mild rigidity with a cogwheel phenomenon was noted.

Despite being able to walk independently, the patient exhibited marked multidirectional instability, particularly upon closing his eyes while maintaining a narrow-based stance. Throughout his hospital stay, involuntary hand movements persisted, at times accompanied by brief episodes of myoclonic jerks. His mental state fluctuated considerably, with abrupt episodes of confusion interspersed with periods of relative alertness.

**Figure 1:**
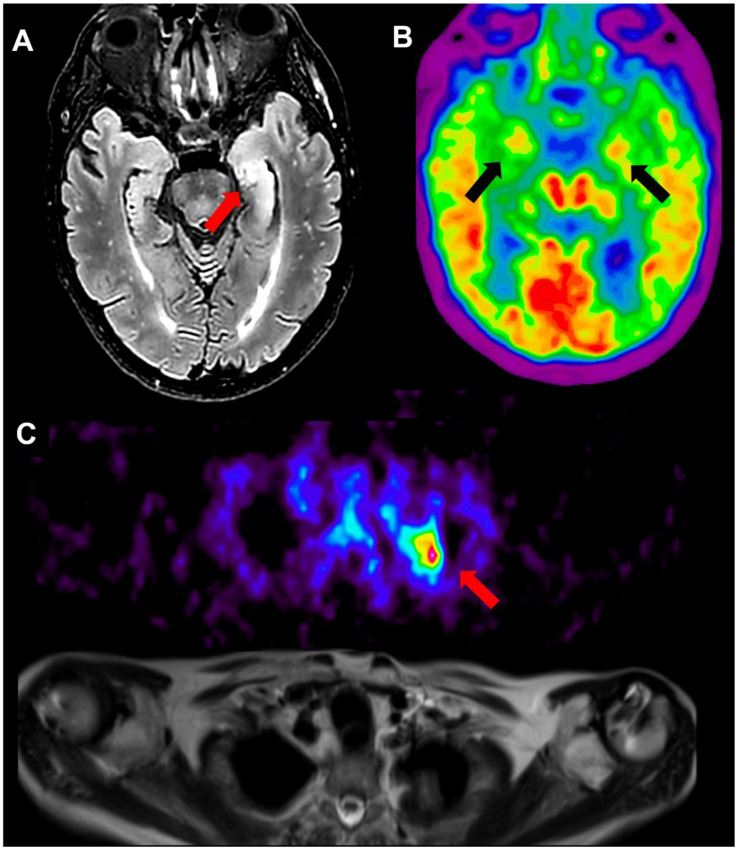
(**A**) T2 Flair MRI demonstrating increased T2 signal in left hippocampus; (**B**) [18F]FDG PET showing bilateral increased uptake in amygdala and portions of hippocampus; (**C**) [18F]FDG PET (**upper**) and T2 Haste MRI (**lower**) showing left apical lung neoplasm.

Brain MRI revealed a FLAIR hyperintensity in the left hippocampus, indicative of inflammation, which was absent in the previous MRI. No diffusion restriction on diffusion-weighted sequences or post-gadolinium enhancement was observed. On brain [18]FDG PET-MRI, global mild cortical hypometabolism was observed, along with concomitant bilateral hypermetabolism of the amygdalae and striatal nuclei. The [18]FDG PET-MRI scan also included the whole body, confirming hypermetabolism of the lung neoplasm (Figure 1)

A lumbar puncture revealed unremarkable physical and chemical findings, with a white blood cell count of 2 cells/µL, protein concentration of 0.46 g/L, and glucose level of 3.3 mmol/L. Cytology did not reveal malignant cells. The serum-CSF protein profile showed no blood–brain barrier disruption nor intrathecal antibody synthesis.

During hospitalization, the patient also developed severe hyponatremia, with sodium levels reaching 118 mmol/L. Daily urinary sodium excretion was 342 mmol/24 h (normal range: 40–220 mmol/24 h). This condition required fluid restriction and treatment with hypertonic saline solution and gradually normalized over two weeks following steroid therapy.

Comprehensive screening for systemic autoimmunity and infectious diseases, including nucleic acid testing for neurotropic viruses in the cerebrospinal fluid (CSF) and a treponemal immunoassay, yielded negative results. Both intracellular (anti-Hu, Yo, Ma2, Ri, GAD) and surface (anti-LGI1, CASPR2, AMPAR, GABAR, NMDAR, DPPX) anti-neuronal antibodies were assessed (Euroimmun cell-based assay). Blood and CSF samples tested positive for anti-LGI1 antibodies. Based on these findings, together with the clinical and radiological features, a diagnosis of LGI1 limbic encephalitis (LE) was established. As a supporting feature, HLA typing at the DRB1 locus revealed heterozygosity for DRB1*07 and DRB*11.

Levetiracetam was added to lacosamide, and 1 g methylprednisolone intravenous boluses were started for five days, with subsequent tapering using oral prednisone (50 mg/day).

At discharge in November 2022, the patient was alert, with preserved working and episodic memory and no focal signs. He still experienced subjective, transient episodes of thinking slowdown, but neither alterations in vigilance nor motor manifestations were observed. In December 2022, a left upper lung lobectomy and hilum-mediastinal lymphadenectomy were performed by video thoracoscopy. The final oncological diagnosis was lung adenocarcinoma (pT2N0, stage T2a) according to the TNM staging system (AJCC 9th edition). Pathology disclosed a moderately differentiated lung adenocarcinoma with a predominantly acinar/papillary pattern, a low degree of necrosis, and PL1 pleural invasion.

Once at home, the patient experienced lack of initiative and apathy, initially responsive to sertraline. In January 2023, more frequent episodes of altered awareness and confusion were noted. After neurological reappraisal and arousing suspicion of relapsing encephalitis, the patient was admitted to the hospital and underwent five sessions of plasma exchange. The treatment proved to be effective, with subjective and clinical improvement of the cognitive disorder and remission of involuntary movements, which allowed for the tapering of antiseizure therapy.

After steroid suspension in June 2023, at follow-up in April 2024, the patient had no focal deficits or involuntary movements. He still complained of apathy, but neither he nor his wife reported any memory deficits. The Mini-Mental State Examination (MMSE) score was 29/30, indicating preserved cognitive function.

At the last follow-up, three years after the onset of encephalitis and following discontinuation of sertraline, the patient had returned to previous activities and reported only mild anxiety and apathy (scoring 1 and 2, respectively, on the Neuropsychiatric Inventory) [7]. Oncological follow-up was negative.

## 3. Discussion

We report a patient diagnosed with LGI1 limbic encephalitis, whose clinical onset preceded the diagnosis of lung adenocarcinoma by two months. Immunotherapy consisting of steroids and plasma exchange, combined with surgical tumor removal, resulted in the complete resolution of neurological manifestations. These findings support a potential paraneoplastic etiology of encephalitis. The case met both clinical and paraclinical criteria for LGI1 autoimmune encephalitis [8] and exhibited several typical features of LGI1 autoimmunity, which we briefly discuss.

In the early phase, the clinical picture of the patient was dominated by brief episodes of unilateral upper limb movements, initially interpreted as focal epileptic seizures and treated with lacosamide, with partial benefit. As more complex clinical manifestations developed, particularly severe anterograde memory deficits, the detection of LGI1 antibodies allowed for the reinterpretation of those episodes as FBDS, which clearly responded to steroidal therapy. In LGI1 encephalitis, FBDS typically precede the onset of cognitive and psychiatric symptoms by some months [9]. They exhibit poor responsiveness to antiseizure medications, but improve more frequently after immunotherapy [10], as observed in our case. Notably, at the last follow-up, while cognitive memory deficits were not significantly detected, persistent psychiatric disturbances—particularly mild anxiety and apathy—were observed. These symptoms have been reported as long-term residual effects following LGI1 encephalitis [11,12,13].

Brain MRI plays a pivotal role in the diagnosis of LGI1 encephalitis; however, up to 20–40% of patients may have a normal brain MRI during the course of the disease [14,15]. Notably, the earliest brain MRI of our case, performed during the phase dominated by FBDS, was unremarkable. In particular, there was no evidence of FLAIR or T1 hyperintensity of caudate nuclei, reported in patients with FBDS [16], or mesiotemporal involvement. However, a subsequent brain MRI, performed after the onset of cognitive deficits, revealed unilateral hippocampal FLAIR hyperintensity. As demonstrated in this case, repeated neuroimaging can help identify radiological signs that support the diagnosis of LE [17,18]. Additionally, the brain [18]FDG PET-MRI showed hypermetabolism in both the mesial temporal structures and the striatal nuclei. This finding highlights the potentially greater sensitivity of PET imaging compared to MRI in detecting alterations in the mesiotemporal lobes and basal ganglia [19,20,21]. Hyponatremia, observed in our patient with levels as low as 118 mmol/L, is a well-recognized feature of LGI1 encephalitis, and is reported in 60–88% of cases. While its exact causes remain incompletely understood, recent studies increasingly support the hypothesis that it is primarily due to SIADH, consistent with prevailing beliefs [22,23,24,25]. All together, these typical features, which are extensively described in patients with LGI1 encephalitis—along with the detection of antibodies through the appropriate assay of both serum and CSF, and the clear response to therapy with steroids and plasma exchange [26]—strongly support the diagnosis. As a further element, the patient was found to be a DRB1*07 carrier, which is present in approximately 90% of patients with anti-LGI1 encephalitis [27].

**Figure 2:**
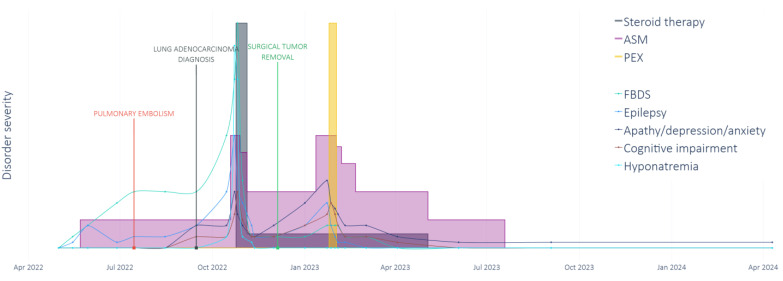
Timeline of clinical manifestations and treatments. AMS: Antiseizure medications; PEX: plasma-exchange sessions; and FBDS: faciobrachial dystonic seizures.

According to the 2021 update of the diagnostic criteria for paraneoplastic neurological syndromes (PNSs) [6], LGI1 antibodies are classified as low risk for paraneoplastic association. Due to the unusual association of LGI1 antibodies with tumors and the unavailability of tissue to be tested for expression of LGI1 antigens, as recommended in atypical cases, we cannot exclude a coincidental association between the encephalitis and the lung adenocarcinoma. Applying the PNS-Care Score [6], our case can be classified as a possible paraneoplastic syndrome, scoring 4 points (3 points for a high-risk phenotype, 0 points for a low-risk antibody, and 1 point for an atypical tumor diagnosed less than 2 years after the onset of neurological manifestations). However, certain features of the case resemble typical findings seen in the management of patients with PNS, supporting a non-coincidental association between the neurological syndrome and the presence of cancer. First of all, there is a strict temporal relationship, which is critical in supporting a diagnosis of PNS, in which usually the cancer is found within 2 years after the onset. Specifically, the neurological symptoms in this case preceded the discovery of lung cancer, which was diagnosed at an early stage of the disease, two months after the onset of FBDS, following a thromboembolic event (Figure 2).

Notably, fewer than 30% of non-small-cell lung cancers are detected at a localized stage (stage I or stage II) [28], but diagnosing cancer in a limited stage is a frequent finding in patients with PNS [29,30,31,32]. It is debated whether this is due to timely screening in the diagnostic work-up of PNS or to the effect of the anti-neoplastic immune response, which underlies the autoimmune mechanisms of PNS. In addition, although the occurrence of lung cancer with LGI1 encephalitis has been reported only anecdotally (see below), non-small-cell lung cancer (including adenocarcinoma) is one of the most frequent tumors reported in PNS. For example, in the large series of 899 cases reported by the PNS Euronetwork in 2010 [29], non-small-cell lung cancer was found in 71 patients (7.9%), after small-cell lung cancer (38.4%), ovarian cancer (10.5%), and breast cancer (9.7%). According to the 2021 PNS criteria, non-small-cell lung cancer is considered a consistent association in patients with neurological syndromes related to Hu, PCA2, Ri, and Ma2 antibodies [6].

LGI1 encephalitis has been rarely reported with paraneoplastic accompaniments. Specifically, as of February 2025, in the largest studies, fewer than 10% of cases are associated with neoplasms. The cancers described are most frequently thymomas, but several other types of tumors are also included, such as prostate cancer and extrapulmonary neuroendocrine tumors [4,22,23,33,34,35]. In some series, isolated cases of lung tumors are reported, without further specification of the histotype and lacking accurate clinical information, making it difficult to distinguish between a coincidental or causal association [11,22,36,37]. Only a few case reports have been described in association with lung tumors, mostly of squamous cell type [35,38,39]. In one of these cases, the causal relationship between LGI1 encephalitis and a squamous lung cancer was supported by the finding that the tumor strongly expressed the LGI1 antigen [39]. In this case, cancer was diagnosed seven months after the onset of encephalitis, which initially manifested with FBDS. To our knowledge, only one other case of LGI1 encephalitis with lung adenocarcinoma has been reported [34], in which the diagnosis was made four months after the onset of limbic encephalitis.

## 4. Conclusions

In conclusion, we report a case of LGI1 limbic encephalitis with a possible paraneoplastic etiology. Based on available data, this association is rare but underscores a potential, albeit uncommon, trigger for the disease. From a practical perspective, the classification of LGI1 antibodies as low-risk according to PNS diagnostic criteria is crucial, as initial screening for occult tumors is generally considered sufficient, and repeat screenings are not recommended [40]. More detailed studies, particularly additional pathological data from further cases, are needed to determine whether this rare association could be causal. This may help refine recommendations for more precise oncological screening or follow-up in a yet-to-be-defined subset of patients.

## Data Availability

The data presented in this study are available on request from the corresponding author due to ethical reasons.
